# A Probabilistic Model of Meter Perception: Simulating Enculturation

**DOI:** 10.3389/fpsyg.2017.00824

**Published:** 2017-05-22

**Authors:** Bastiaan van der Weij, Marcus T. Pearce, Henkjan Honing

**Affiliations:** ^1^Music Cognition Group, Amsterdam Brain and Cognition, Institute for Logic, Language, and Computation, University of AmsterdamAmsterdam, Netherlands; ^2^Music Cognition Lab, School of Electronic Engineering and Computer Science, Queen Mary University of LondonLondon, United Kingdom

**Keywords:** rhythm, cognition, meter perception, predictive coding, enculturation, computational modeling

## Abstract

Enculturation is known to shape the perception of meter in music but this is not explicitly accounted for by current cognitive models of meter perception. We hypothesize that the induction of meter is a result of predictive coding: interpreting onsets in a rhythm relative to a periodic meter facilitates prediction of future onsets. Such prediction, we hypothesize, is based on previous exposure to rhythms. As such, predictive coding provides a possible explanation for the way meter perception is shaped by the cultural environment. Based on this hypothesis, we present a probabilistic model of meter perception that uses statistical properties of the relation between rhythm and meter to infer meter from quantized rhythms. We show that our model can successfully predict annotated time signatures from quantized rhythmic patterns derived from folk melodies. Furthermore, we show that by inferring meter, our model improves prediction of the onsets of future events compared to a similar probabilistic model that does not infer meter. Finally, as a proof of concept, we demonstrate how our model can be used in a simulation of enculturation. From the results of this simulation, we derive a class of rhythms that are likely to be interpreted differently by enculturated listeners with different histories of exposure to rhythms.

## 1. Introduction

In a variety of settings, perception appears to be tuned to statistical properties of the environment. It has for example been found that certain properties of neuron receptive fields in early visual processing (Olshausen and Field, 1996) and early auditory processing (Smith and Lewicki, 2006) emerge from information theoretically efficient learning algorithms trained respectively on natural images or sounds. Such tuning, it has been suggested, happens both on an evolutionary time-scale through gradual adaptation, and on an ontogenetic time scale, through brain plasticity (Clark, 2013).

The perception of meter in music appears to be shaped by cultural differences in musical conventions. Exposure to rhythmically different music has been shown to influence perception from an early age (Hannon and Trehub, 2005a,b), but such shaping possibly continues into adulthood (Creel, 2011, 2012). In the current paper, we hypothesize that considering meter perception from the perspective of *predictive coding* (Rao and Ballard, 1999; Friston, 2005; Clark, 2013) can help to understand how meter perception is shaped by one's environment.

Rhythm is an important component of music traditions all over the world (Savage et al., 2015). When listening to rhythms, onsets in the rhythm are perceived relative to a periodic and hierarchically organized framework of beats (Honing, 2013). This mental framework, called meter, is induced in the mind of the listener by the rhythm. The relation between rhythm and meter is complex. For a meter to be perceived, not every beat in the meter needs to coincide with onsets in the rhythm. In many cases, listeners can, through conscious effort, alter their metrical interpretation of a rhythm. At the same time, not every meter is equally easy to hear in every rhythm. Meter, once induced, tends to show a certain resistance to change. Therefore, meter perception is a fundamentally incremental process (Longuet-Higgins and Steedman, 1971): the same rhythmic passage can sound different depending on the meter induced by the rhythm preceding the passage (Honing, 2013).

The organizing structure of meter is commonly described as a hierarchy of pulses, yielding a periodic pattern of metrical accents varying in salience at different points in time. Metrical accent, or metrical salience, is commonly treated as a proxy for temporal expectation, or the probability of an event onset at a particular pulse (Palmer and Krumhansl, 1990). By investigating a corpus of Western classical music, Palmer and Krumhansl (1990) found that the distribution of onsets over different positions relative to the meter reflected theoretical descriptions of metrical hierarchy (Lerdahl and Jackendoff, 1983). Using a goodness-of-fit paradigm, Palmer and Krumhansl (1990) found that temporal *expectations* of North-American listeners also reflect metrical hierarchy, although musicians showed evidence of deeper hierarchical differentiation than non-musicians. Based on these findings, Palmer and Krumhansl (1990) suggested that composers communicate meter to listeners through the distribution of onsets at different metrical positions. Listeners, in turn, acquire their knowledge about meter through the distribution of onsets over metrical positions in the music they are exposed to.

More recent work has addressed the question of whether hierarchical organization of onset distributions is a general property of rhythmic organization or whether it is specific to Western classical music and related styles. Holzapfel (2015), for instance, found that in traditional Turkish makam music, the distribution of onsets is modulated by the specific *usul*—a type of rhythmic mode, corresponding in some ways to meter—underlying a piece. Furthermore, the distribution of onsets within one usul in Turkish makam music does not always exhibit hierarchical organization. London et al. (2016) found that peaks in onset distributions in a corpus of Malian drumming recordings are not periodically spaced. London et al. (2016) conclude that in makam music and Malian drumming, distributions of onsets do reflect metrical structure, but this structure is not always isochronous or strictly hierarchical.

London et al. (2016) point out that their and Holzapfel (2015) results question a basic assumption made by many computational models, as well as empirical studies, namely that metrical accent is equivalent to the likelihood of an onset. A more likely alternative is that metrical expectations are derived from extensive exposure to a musical idiom, by which, beyond distributions of onsets and style-specific, stereotypical rhythmic patterns associated with certain meters are learned.

Consistent with this suggestion, an increasing number of empirical studies show that rhythm perception is affected by enculturation (cf. Morrison and Demorest, 2009). For example, Bulgarian or Macedonian adults are better in detecting metrical violations in meters with a non-isochronous *tactus* level—the level of beat that listeners are most likely to tap along with—(e.g., 5/8 or 7/8) than North-American listeners (Hannon and Trehub, 2005a). This effect appears to be specific to complex meters to which the listeners have been exposed (Hannon et al., 2012).

There have also been a number of observations in the ethnomusicological literature suggesting that individuals from different cultures perceive rhythms differently. For example, during field work in the Bolivian Andes, while studying Easter songs from Northern Potosí, (Stobart and Cross, 2000) realized that while they had assumed many of the tunes where indisputably anacrustic (i.e., a rhythm starting on an off-beat), the local populations appeared to perceive them as beginning on a downbeat. Another example is provided by rhythms from West-African Sub-Saharan musical cultures, which are characterized by a great deal of metrical ambiguity (Locke, 1982). In particular, many of these rhythms can be interpreted as having a binary or ternary pulse. While individuals from West-African cultures appear to perceive both pulses with equivalent ease, it can take great effort for Western listeners to hear the ternary pulse in some of these rhythms.

The idea that perception, in general, is shaped by statistical properties of the environment is not new (e.g., Barlow, 1961). However, it recently has been developed into a framework which has been argued to bear the promise of providing an overarching theory of perception (Clark, 2013). Under the name of predictive coding (Rao and Ballard, 1999), this framework firmly grounds perception in prediction, based largely on previous sensory experience. In fact, the theory proposes that the brain's primary occupation is to explain sensory input using hierarchical generative models gleaned from previous experience (Clark, 2013). Such models are realized in a hierarchical organization of layers. The lowest layer in the hierarchy represents sensations received directly from the senses. Through feed-forward connections, information travels upward in the hierarchy. Meanwhile, layers higher up in the hierarchy attempt to predict information, propagated by layers below. These predictions are cast to lower layers through feedback connections. Successful prediction cancels out the upward propagation of information. As a result, only *prediction error*, information that higher layers failed to predict, propagates upwards in the hierarchy. Based on prediction error, layers gradually adapt their processing characteristics in a way that minimizes prediction error with respect to layers lower in the hierarchy. By this process of adaptation, the hierarchy of layers is gradually shaped into a *generative* model of sensations, where layers higher up in the hierarchy track causes in the external world that underlie the received sensations (Friston, 2005). From an information-theoretic point of view, the resulting coding scheme is highly efficient: the more accurate the top-down predictions, the less bottom-up information is left to be processed.

We propose a predictive coding account of meter perception that involves statistical learning of musical rhythms and generation of probabilistic expectations for event timings. Meters are modeled as distinct causes underlying the musical surface. Inferring the underlying meter from rhythm allows the rhythm to be related to rhythms previously heard in that meter, which may help prediction performance. Enculturation is modeled by estimating the parameters of the generative model on a corpus of quantized rhythms annotated with meter. Since the model learns the statistical properties of rhythms through exposure and performs metrical inference based on these, it has the potential to simulate enculturation effects in meter perception.

The paper is organized in six sections. In the remaining part of the current section, Section 1.1 develops an account of meter perception based on predictive coding, while Section 1.2 discusses relevant work in computational modeling of music perception. Section 2 presents the probabilistic model of meter perception in detail, concluding with a set of behaviors we expect the model to exhibit. Section 3 presents the methods used in a series of simulations designed to test these behaviors, while Section 4 presents the results of the simulations. Section 5 discusses the results in the context of the existing literature and includes implications for future research.

### 1.1. Meter perception as predictive coding

The dynamic interaction of top-down and bottom-up processing postulated by predictive coding is reminiscent of dynamic interaction of bottom-up meter-induction and top-down influence exerted by the induced meter, as pointed out by Vuust and Witek (2014).

The hypothesis we explore in this paper is that predictive coding can explain how meter perception is influenced by enculturation. To explore the consequences of this idea, we present a probabilistic model of meter perception, based on an empirical Bayes scheme. Empirical Bayes schemes describe how generative systems, such as the generative models posited by predictive coding, are updated by experience (Friston, 2005). We model meters as virtual causes underlying the rhythmic surface: a meter imposes constraints the likelihood of rhythms. A listener commanding an appropriate generative model reflecting this relationship (i.e., how rhythms are generated from meters), can, when presented only with a rhythmic surface, infer the underlying meter. This process of inferring underlying causes (meters) of experienced sensations (rhythms) involves inverting the generative model of those sensations (which are the end-product of the generative process). We hypothesize that interpreting the rhythm in the context of an inferred meter will reduce the discrepancy between predicted and experienced sensations. In other words, inferring meter makes the rhythm more predictable.

The generative model includes prior expectations, obtained from previous experience, about which metrical categories are likely to occur in general. For example, meters with non-isochronous pulses (“complex” meters) are relatively uncommon in Western-European music, but much more common in music from the Balkans and Eastern Mediterranean region. Listeners from these regions may be more likely to interpret a rhythm in a meter with non-isochronous pulses than listeners from Western Europe. These kind of prior biases might underlie the findings of Hannon and Trehub (2005a) mentioned in the previous section.

Metrical categories favored by prior biases entail expectations regarding the surface structure of rhythms. As bottom-up evidence from the rhythm begins to flow in, these (top-down) expectations are either confirmed or violated. Prediction error results from a violation of the top-down expectations by the incoming evidence. To reduce prediction error, the listener revises their metrical interpretation of the rhythm, which in turn alters the flow of top-down predictions. A predictive coding perspective of meter perception thus posits a dynamic interplay between bottom-up evidence and top-down expectations.

Crucially, both prior biases toward certain meters and the dependencies between meter and the rhythmic surface—which rhythms can be generated by a certain meter—are the result of previous exposure. The generative model in the mind of the listener underlying these representations is carved out by previous experience in predictive processing of rhythmic signals. Since the statistical properties of rhythms vary between styles (e.g., Holzapfel, 2015; London et al., 2016), the processing biases of listeners with significant differences in their exposure to musical styles are likely to vary as well.

### 1.2. Related work

Our approach in some respects resembles other recent probabilistic models, in particular a generative model presented by Temperley (2007). Temperley (2007, ch. 2) models meter perception as probabilistic inference on a generative model whose parameters are estimated using a training corpus. Meter is represented as a multi-leveled hierarchical framework, which the model generates level by level. The probability of onsets depends only on the metrical status of the corresponding onset time. Temperley (2009) generalizes this model to polyphonic musical structure, and introduces a metrical model that conditions onset probability on whether onsets occur on surrounding metrically stronger beats. This approach introduces some sensitivity to rhythmic context into the model. In later work, Temperley (2010) evaluates this model, the *hierarchical position model*, and compares its performance to other metrical models with varying degrees of complexity. One model, called the first-order metrical position model, was found to perform slightly better than the hierarchical position model, but this increase in performance comes at the cost of a higher number of parameters. Temperley concludes that the hierarchical position model provides the best trade-off between model-complexity and performance.

In a different approach, Holzapfel (2015) employs Bayesian model selection to investigate the relation between usul (a type of rhythmic mode, similar in some ways to meter) and rhythm in Turkish makam music. The representation of metrical structure does not assume hierarchically organization, allowing for arbitrary onset distributions to be learned. Like the models compared by Temperley (2010), this model is not presented explicitly as a meter-finding model, but is used to investigate the statistical properties of a corpus of rhythms.

The approach presented here diverges from these models in that it employs a general purpose probabilistic model of *sequential* temporal expectation based on statistical learning (Pearce, 2005) combined with an integrated process of metrical inference such that expectations are generated given an inferred meter. The sequential model is a variable-order metrical position model. Taking into account preceding context widens the range of statistical properties of rhythmic organization that can be learned by the model. In particular, the model is capable of representing not only the frequency of onsets at various metrical positions, but also the probability of onsets at metrical positions conditioned on the preceding rhythmic sequence. The vastly increased number of parameters of this model introduces a risk of *over-fitting*; models with many parameters may start to fit to noise in their training data, which harms generalization performance. However, we employ sophisticated smoothing techniques that avoid over-fitting (Pearce and Wiggins, 2004). Furthermore, we to some extent safe-guard against over-fitting by evaluating our model using cross-validation.

## 2. The probabilistic model

In this section and the sections that follow, we use the words metrical category and metrical interpretation in a specific sense. *Metrical categories*, denoted by *m*, represent different metrical frameworks in which rhythms can be interpreted. Metrical categories correspond directly to time signatures taken from scores. Each metrical category has an associated *period*, denoted by *T*_*m*_. The period is encoded as a discrete number representing the duration of one bar of *m* in basic quantized units of time (see Section 2.1). The *phase* parameter, ϕ, encodes how a metrical category aligns with the rhythmic surface. More precisely, ϕ encodes the time-interval between the downbeat of the first bar and the time point marked by zero in the encoding of the rhythmic pattern. Together, a metrical category and phase form a *metrical interpretation*.

The approach described below deals not with real audio signals. Instead, the musical surface is represented as a sequence of events. Each event corresponds to a note, as it might be found in a musical score. The *n*th event in a sequence is denoted by *e*_*n*_. A sequence of events, starting at event *n* and ending at event *m* is denoted by ${\text{e}}_{n}^{m}$. Section 2.1 provides more details the representation of rhythmic patterns.

Predictive coding postulates internal generative models reflecting the causal structure of the external world. In analogy to this, we model meter perception as the inversion of a generative model of rhythms. Enculturation through exposure to rhythms is modeled by deriving the parameters of the generative model from a corpus of rhythms annotated with metrical interpretation. During listening, the metrical category underlying a given rhythm is generally not known to the listener. Instead, it has to be inferred from rhythmic surface, which is assumed to result from the generative model. The likelihood of a metrical interpretation given an observed rhythm (i.e., a sequence of events) can be inferred from the generative model through the application of Bayes' formula, as shown in Equation (1).

(1) $$\begin{array}{r} \overset{\text{posterior}}{\overset{︷}{p\left(m,\phi |{\text{e}}_{0}^{n}\right)}}=\frac{\overset{\text{likelihood}}{\overset{︷}{p\left({\text{e}}_{0}^{n}|m,\phi \right)}}\overset{\text{prior}}{\overset{︷}{p\left(m,\phi \right)}}}{\underset{\text{evidence}}{\underset{︸}{p\left({\text{e}}_{0}^{n}\right)}}}. \end{array}$$

Two factors play a role in calculating the likelihood of a metrical category: The *a priori* likelihood of the metrical category itself, operationalized here as the metrical category's conventionality. In Equation (1), this distribution is labeled *prior*. The other factor is the likelihood of the rhythmic pattern given a certain metrical structure. In Equation (1), this function is labeled *likelihood*. The distribution over metrical interpretations inferred from the observed events is called the *posterior* distribution. The factor labeled *evidence* in Equation (1) is a constant with respect to metrical interpretation. It ensures that the distribution sums to unity.

**Figure 1:**
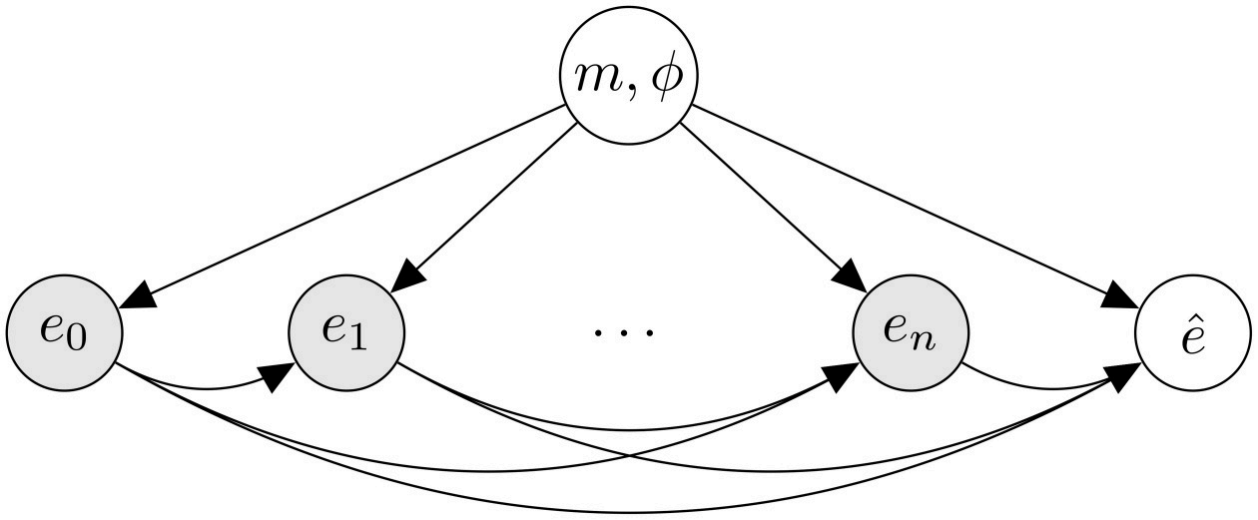
**Conditional dependency relations assumed by the model between its probabilistic variables visualized as a graphical model (Bishop, **2007**, ch. 8)**. Shaded nodes represent observed variables, unshaded nodes represent unobserved, *hidden* variables. Each node in the graph is associated with a discrete probability distribution. If one or more arrows terminate at a node, its associated probability distribution is conditioned on the node(s) that the arrows originate from. Nodes labeled *e*_*n*_ represent musical events indexed by *n*. The hidden variable at the top represents a metrical interpretation. The hidden variable labeled $\hat{e}$ represents a predicted subsequent event.

The proposed generative model is illustrated in Figure 1. To generate a rhythm, a metrical category is first generated from a distribution, *p*(*m*), reflecting the prior likelihood of metrical categories. Next, a phase is sampled from a uniform distribution over a range of discrete phases allowed in *m*. From a model associated with the selected metrical category, events are then generated in an incremental fashion. As can be seen in Figure 1, the likelihood of an event is conditioned on underlying metrical category and preceding events.

Equation (1) can be expanded into the incremental and recursive equation shown in Equation (2). This equation expresses the posterior distribution given all events as proportional to the product of the likelihood of the last event, *e*_*n*_ and the posterior given all but the last event, ${\text{e}}_{0}^{n-1}$. Inferring the posterior incrementally after each event by refining the posterior that resulted from the previous events can be interpreted intuitively as the listener integrating the (bottom-up) information provided by each event into their (top-down) beliefs about the underlying metrical category. Note that the evidence normalization constant has been omitted for clarity.

(2) $$\begin{array}{r} \overset{\text{per-event posterior}}{\overset{︷}{p\left(m,\phi |{\text{e}}_{0}^{n}\right)}}\propto \begin{array}{cc} \overset{\text{per-event likelihood}}{\overset{︷}{p\left(e_{n}|m,\phi ,{\text{e}}_{0}^{n-1}\right)}}\overset{\text{updated prior}}{\overset{︷}{p\left(m,\phi |{\text{e}}_{0}^{n-1}\right)}}\; & \text{if }n>0,\\ p\left(e_{n}|m,\phi \right)p\left(m,\phi \right)\; & \text{else}. \end{array} \end{array}$$

To infer the posterior distribution over metrical interpretations, Equation (2) is evaluated for a set of possible metrical interpretations. This set is constrained to include only metrical categories that occur in the model's training data. The number of different phases considered per metrical category depends on the period of the category, *T*_*m*_.

To evaluate Equation (2), two probability distributions need to be approximated: the prior distribution over metrical interpretations, *p*(*m*, ϕ), and the likelihood function *p*(${\text{e}}_{0}^{n}$|*m*, ϕ). We discuss both in the following paragraphs.

First, we consider estimating the prior, which uses supervised learning from a corpus of rhythms labeled with metrical category. The parameters of the distribution defining the *a priori* likelihood of metrical categories (not phases), *p*(*m*), are set to their maximum likelihood estimate, namely the relative frequency of occurrence of a metrical category in the empirical training data.

(3) $$\begin{array}{r} p\left(m\right)=\frac{N_{m}}{N}, \end{array}$$

where *N*_*m*_ is the number of times *m* was observed in the training data and *N* is the total number of training examples (rhythms) in the training data.

The prior distribution over metrical interpretations (i.e., the joint distribution over phase and metrical category) is defined as follows:

(4) $$\begin{array}{r} p\left(m,\phi \right)=\frac{p\left(m\right)}{\overset{m^{\prime }}{\sum }T_{m^{\prime }}p\left(m^{\prime }\right)}. \end{array}$$

Each metrical interpretation is assigned a probability proportional to the probability of its category. This definition entails a reweighing of metrical categories to compensate for the duration of their periods; it prevents meters with long periods (many possible phases) from being at a disadvantage due to the uniform spreading out of their probability over a large number of phases.

Second, we consider estimating the likelihood. Calculating the likelihood of an observed rhythm given a hypothesized metrical interpretation involves two steps: First, the rhythm under consideration is *interpreted* in a hypothesized metrical interpretation specified by *m* and ϕ. Interpretation is operationalized in the present model as converting the events in the rhythm into a sequence of symbols encoding the position of each event relative to the beginning of the bar in which it occurs under the currently considered metrical interpretation. The details of this conversion are discussed in Section 2.3. Second, the likelihood of the resulting sequence of symbols is estimated using an unsupervised probabilistic model trained on metrically interpreted rhythms in the training corpus annotated with the same metrical category, *m*. The likelihood that a rhythm is generated by given metrical interpretation thus becomes the likelihood of the sequence of symbols resulting from metrically interpreting the event onset times in the rhythm. The likelihood of the metrically interpreted rhythm, in turn, is determined on the basis of a corpus of rhythms belonging to the same metrical category.

Equation (2) decomposes the likelihood function into the product of the per-event likelihoods, i.e., the likelihood of each (metrically interpreted) event given the sequence of (metrically interpreted) preceding events. In the present work, IDyOM (Pearce, 2005) is used to approximate the per-event likelihood function.

IDyOM is a flexible modeling framework based on variable-order Markov modeling combined with a multiple-viewpoint system for music prediction (Conklin and Witten, 1995). It was designed for modeling dynamically changing auditory expectations, based on long-term and short-term statistical learning, which evolve as a piece of music unfolds. Empirical research has demonstrated that IDyOM accurately simulates listeners' predictive processing of melody in many perceptual tasks involving pitch expectation (Pearce, 2005; Pearce et al., 2010; Omigie et al., 2012, 2013), uncertainty (Hansen and Pearce, 2014), segmentation (Pearce et al., 2010) and emotional response (Egermann et al., 2013; Gingras et al., 2015).

Section 2.3 describes how our model is implemented on top of IDyOM. While the present model does not make use of the full range of modeling opportunities that the multiple-viewpoint approach has to offer, presenting the model as an extension of IDyOM highlights the continuity between the two probabilistic modeling approaches.

Aspects of multiple viewpoint systems and IDyOM relevant to the present model are introduced in Section 2.1 and Section 2.2. Our treatment of this topic is far from complete; for a complete overview, we refer the reader to Conklin and Witten (1995) and Pearce (2005).

### 2.1. Representation of rhythmic patterns

Multiple viewpoint systems represent the musical surface as a sequence of multi-dimensional datapoints encoding basic attributes of musical events, such as pitch, onset time and duration. These basic attributes of events are accessed through *viewpoints*. A viewpoint maps sequences of events, rather than individual events, to an element of its corresponding *type*, τ. The set of all possible elements of a type τ is called the *alphabet* of τ and is denoted by [τ]. A viewpoint function may be undefined for some sequences of events. The inter-onset-interval viewpoint, for example, is undefined for the sequence **e**00, which consists of only a single event, *e*_0_. Hence, a viewpoint is defined by a partial function that maps sequences of events to elements of a type

$$\begin{array}{l} \Psi_{\tau }:\zeta^{*}⇀\left[\tau \right], \end{array}$$

where the symbol ζ^*^ denotes the set of all possible sequences of events.

A distinction between two types of viewpoints is made. A *basic viewpoint* simply returns one of the basic attributes of the last event in the sequence to which it was applied (i.e., a projection function). The alphabet of a basic viewpoint is determined by the set of values of its corresponding attribute observed in the training corpus (see Section 2.2). A *derived viewpoint* derives more abstract attributes from one or more basic attributes of one or more basic events. Its alphabet can be derived from the alphabets of the basic viewpoints that the viewpoint is derived of. The inter-onset-interval viewpoint and metrical viewpoints introduced in Section 2.3 are examples of derived viewpoints. For derived viewpoints, multiple different sequences of events may map to the same element.

The function Φ_τ_ returns the sequence of viewpoint elements of type τ obtained by applying the viewpoint function Ψ_τ_ incrementally to to all prefixes of the sequence in order of increasing length:

$$\begin{array}{l} \Phi_{\tau }\left({\text{e}}_{0}^{n}\right)=\begin{array}{cc} \Phi_{\tau }\left({\text{e}}_{0}^{n-1}\right)\Psi_{\tau }\left({\text{e}}_{0}^{n}\right)\; & \text{if}\Psi_{\tau }\left({\text{e}}_{0}^{n}\right)\neq ⊥\text{,}\\ \Phi_{\tau }\left({\text{e}}_{0}^{n-1}\right)\; & \text{else}, \end{array} \end{array}$$

where ⊥ is a symbol indicating that the viewpoint is undefined for the given sequence of events.

The model introduced here makes use of a single basic viewpoint, namely on, returning the onset attribute of the last event in a sequence, and a set of derived metrical viewpoints. The alphabet of onset, [on], contains natural numbers that encode the temporal position of a note as an integer-multiple of basic quantized units. To obtain a finite, meaningful alphabet for on, the onset alphabet is constructed online by adding the set of inter-onset intervals encountered in the training data to the onset of the previous event.

### 2.2. Predicting musical events

Predicting sequences of musical events in IDyOM requires specifying a set of viewpoints, τ_0_, τ_1_, ⋯ , τ_*n*_, on which to base predictions. A predictive model is associated with each of these viewpoints. Each predictive model is trained on the set of symbol sequences obtained by applying the associated viewpoint function Φ_τ_ to all event sequences in the training corpus. To approximate the predictive distribution for a future event, $p\left(\hat{e}|{\text{e}}_{0}^{n}\right)$, given a sequence of preceding events ${\text{e}}_{0}^{n}$, the function Φ_τ_ is applied, once for each of the specified viewpoints, to ${\text{e}}_{0}^{n}$ to obtain a set of sequences of viewpoint elements.

The per-viewpoint predictions, $p_{\tau }\left(\Psi_{\tau }\left(\hat{e}\right)|\Phi_{\tau }\left({\text{e}}_{0}^{n}\right)\right)$ are then combined into a single event prediction, using a mechanism that involves a weighted geometric mean. Some subtleties are involved in converting the predictive distributions to a single domain so that they can be combined (Pearce, 2005, ch. 7). These need not concern us, as the model proposed here only uses a single viewpoint to predict a single attribute of the event representation (although it could be extended in the future to use multiple viewpoints).

IDyOM thus reduces the challenge of estimating $p\left(\hat{e}|{\text{e}}_{0}^{n}\right)$ to the parallel prediction of symbol sequences by estimating $p_{\tau }\left(\Psi_{\tau }\left(\hat{e}\right)|\Phi_{\tau }\left({\text{e}}_{0}^{n}\right)\right)$ for each viewpoint τ_0_, τ_1_, ⋯ , τ_*n*_. The (domain-general) method employed by IDyOM for predicting symbol sequences is based on a data-compression scheme called prediction by partial matching (PPM), introduced by Cleary and Witten (1984). Pearce and Wiggins (2004) provide an overview of various modifications and improvements to the original PPM scheme that have been proposed over the years, and compare their performance using an information-theoretic performance measure (see Section 2.4). IDyOM implements multiple prediction schemes and furthermore allows predictions to be based on two separate models: a long-term model trained on a corpus of training data and a short-term model trained, online, on only the current sequence of events. In our simulations, we use only a long-term model (see Pearce et al., 2005), employing a PPM^*^ scheme using method C (Moffat, 1990) for calculating escape probabilities and adapted to use interpolated smoothing—the configuration Pearce and Wiggins, 2004 found to yield the best results for a long-term model. A parameter called model order-bound parameter limits the amount of previous events taken into account in the predicting the next event, $\hat{e}$: An order-bound of *b* means that it is assumed that $p\left(\hat{e}|{\text{e}}_{0}^{n}\right)\approx p\left(\hat{e}|{\text{e}}_{n-b}^{n}\right)$. While Pearce and Wiggins (2004) found that an unbounded model order worked best, the present paper presents results for varying model order-bounds of up to four.

### 2.3. Metrical viewpoints, metrical models, and metrical inference

The per-event likelihood function in Equation (2) is a predictive distribution that, based on events observed so far and a hypothesized metrical interpretation, specified by *m* and ϕ, predicts the next event. This relies on interpreting the sequence of events in the given metrical interpretation and estimating the likelihood of the resulting sequence of symbols given a predictive model of such sequences in the provided metrical category. Interpretation of a rhythm in a specific metrical interpretation is achieved in IDyOM through the introduction of a set of *metrical viewpoints*. Metrical viewpoints transform a sequence of absolute onset times into a sequence of symbols that depend on the metrical interpretation implemented by the viewpoint.

The general form of a metrical viewpoint τ_*m*,ϕ_ is

$$\begin{array}{l} \Psi_{\tau_{m,\phi }}\left({\text{e}}_{0}^{n}\right)=f\left(m,\phi ,{\text{e}}_{0}^{n}\right), \end{array}$$

where *f* is a function that implements the metrical interpretation given a phase and metrical category.

The present model uses a simple metrical interpretation function that returns the *metrical position* of an onset. This function makes few assumptions about the structural organization of meter, and can accommodate complex, non-isochronous meters. The metrical position of an onset is defined as its position relative to the period and phase of an interpretation. The general definition of the resulting metrical position viewpoint, mp, is given below

$$\begin{array}{l} \Psi_{{\text{mp}}_{m,\phi }}\left({\text{e}}_{0}^{n}\right)=\left(\Psi_{\text{on}}\left({\text{e}}_{0}^{n}\right)-\phi \right)\;\mathrm{mod}\;T_{m}, \end{array}$$

where the viewpoint on is a basic viewpoint that returns the onset of the last event in a sequence of events.

One metrical viewpoint is created for each metrical interpretation considered by the model by instantiating *m* and ϕ to a specific value.

The alphabet of the mp viewpoint is given by

$$\begin{array}{l} \left[{\text{mp}}_{m,\phi }\right]=\left\{0,1,\cdots \;,T_{m}-1\right\}. \end{array}$$

Using metrical viewpoints, metrical inference can be implemented on top of the standard IDyOM machinery, with one important caveat: the predictive model of a metrical viewpoint, τ_*m*,ϕ_ is trained only on those sequences in the training data that have been annotated with metrical category *m*. Hence, the predictability of a metrically interpreted rhythm depends only on rhythms previously observed in the corresponding metrical category.

One further subtlety needs to be addressed to complete the model. Note that the per-viewpoint predictive distributions mentioned in Section 2.2 are defined over a viewpoint's alphabet [τ]. In order to predict the onset of the next event this alphabet needs to be mapped back to the alphabet of the onset viewpoint, [on]. However, any metrical position in [mp] theoretically corresponds to an infinite number of periodically spaced onset times. To be able to generate predictions for *specific* onset times, and for metrical inference to work correctly, it is necessary that the alphabet of a metrical viewpoint maps to unique onset times. This can be achieved by *linking* the metrical position viewpoint to another metrical viewpoint, which encodes the distance in bars between the last event and the predicted event.

The equation below defines the bar distance viewpoint, bd in terms of an intermediate metrical viewpoint, bn (bar number), which calculates the number of bars elapsed between time zero and the onset of the last event.

$$\begin{array}{l} \Psi_{{\text{bd}}_{m,\phi }}\left({\text{e}}_{0}^{n}\right)=\Psi_{{\text{bn}}_{m,\phi }}\left({\text{e}}_{0}^{n}\right)-\Psi_{{\text{bn}}_{m,\phi }}\left({\text{e}}_{0}^{n-1}\right), \end{array}$$

where metrical viewpoint bn is defined as

$$\begin{array}{l} \Psi_{{\text{bn}}_{m,\phi }}\left({\text{e}}_{0}^{n}\right)=\text{integer}\left(\frac{\left(\Psi_{\text{on}}{\text{e}}_{0}^{n}-\phi \right)}{T_{m}}\right). \end{array}$$

A linked viewpoint is a special case of a derived viewpoint composed of a number of constituent viewpoints. The elements of linked viewpoints are tuples containing the values of the constituent viewpoints. A linked viewpoint composed of τ_1_, ⋯ , τ_*n*_ is denoted by τ_1_ ⊗ ⋯ ⊗ τ_*n*_, its alphabet is given by the Cartesian product of the constituent viewpoints' alphabets: [τ_1_] × ⋯ × [τ_*n*_].

The linked metrical viewpoint used in our simulations is denoted by mp ⊗ bd, and encodes metrical position and distance in bars between the penultimate and last event. Elements in the alphabet of this viewpoint have a one-to-one correspondence to elements in [on].

To summarize: metrical viewpoints and separate predictive models per metrical category enable using IDyOM to estimate the per-event likelihood function in Equation (2). In this model, the likelihood of a metrical interpretation *m* depends on the predictability of the sequence of symbols that results from interpreting the rhythm in that metrical interpretation. This predictability in turn depends on the set of rhythms previously observed in *m*.

### 2.4. Expectation and information content

We have focussed our discussion so far on the issue of inferring a posterior distribution over metrical interpretations. In order to calculate prediction error, it is necessary to derive the predictive distribution over future note onsets given a preceding rhythmic context and an inferred meter.

To estimate prediction error, we look at the amount of information communicated by each observation. Although it is sometimes referred to as cross-entropy (e.g., Manning and Schütze, 1999, ch. 2), we call this quantity the *information content* (MacKay, 2003) of an event. Information content is defined as the negative logarithm of the likelihood of observing the next event given the predictive distribution conditioned on the sequence of events observed so far:

(5) $$\begin{array}{r} h\left(\hat{e}|{\text{e}}_{0}^{n}\right)=-\log_{2}p\left(\hat{e}|{\text{e}}_{0}^{n}\right). \end{array}$$

In an information-theoretic sense, this quantity is equivalent to prediction error. An unlikely (unexpected) event results in a high prediction error, signaled by high information content. Conversely, a likely event results in a low prediction error, signaled by low information content.

The predictive distribution corresponds to the probability distribution associated with the hidden variable labeled $\hat{e}$ in the graphical model in Figure 1. This distribution is obtained from the generative model by marginalizing out meter and phase from the posterior distribution inferred from the preceding events:

(6) $$\begin{array}{r} p(\hat{e}|e_{0}^{n})=\sum^{m}\sum^{\phi }p(\hat{e}|m,\phi ,e_{0}^{n})p(m,\phi |e_{0}^{n}), \end{array}$$

where the summation over meters sums over all metrical categories considered by the model, *m* ∈ *M*, and the summation over phases sums over all possible phase of category *m*, ϕ ∈ {0, 1, ⋯ , *T*_*m*_ − 1}.

Equation (6) shows that the prediction of the onset of the next event is subject to top-down influence from the distribution over metrical interpretations inferred from bottom-up information from the events observed so far.

### 2.5. Hypotheses

We expect an accurate computational model of human meter perception to show certain patterns of behavior. First, we expect it to be able to infer meters that agree with the time signatures in notated scores (Longuet-Higgins and Lee, 1982; Temperley, 2004). Second, we argued that the metrical knowledge, acquired by listeners through exposure to a musical idiom, is characterized not only by the distribution of onsets over metrical positions, but also by the probabilistic properties of how rhythms in particular meters sequentially unfold. Thus, we expect that a model that can learn such properties will lead to increased performance in finding time signatures notated in scores compared to a similar model that does not learn these properties. Third, we argued above that categorizing rhythms into metrical categories can plausibly be regarded as a strategy to reduce prediction error for those rhythms. Therefore, we expect that our model will show better performance in predicting the timing of musical events than a comparable model that is agnostic of meter. Fourth, we expect that our model will simulate enculturation by showing sensitivity to the statistical properties of the rhythms it was trained on. A model trained on rhythms with similar statistical properties as the rhythms it is evaluated on will perform better than a model that was trained on rhythms with different statistical properties. If the statistical properties of rhythms originating from two cultures with different cultural practices regarding rhythm are sufficiently different, we expect that a model trained on rhythms from the same culture as the rhythms it is evaluated on will outperform a model trained on rhythms from a culture with different rhythmic practices. We evaluate these expectations in Sections 3 and 4.

## 3. Methods

### 3.1. Resolution of onset time and phase

For reasons of computational efficiency, the resolution the phase parameter of metrical interpretations is restricted to sixteenth notes. This means that, for example, in the 3/4 category twelve different phases are possible (since the duration of one 3/4 bar is twelve sixteenth notes). Since all onset times in rhythms used in this study encode distance from the beginning of the first bar in the annotated meter, the correct phase of a rhythm can be represented under any phase resolution. The representation of rhythms in a phase of zero does not influence the evaluation: as far as the model is concerned, all phases are initially equally likely since the prior distribution over phase is uniform. The presence of 32th notes and 16th-note triplets in the training data requires that onset times are represented as integer multiples of symbolic units corresponding to 96th notes.

### 3.2. Training data

Except for one artificially constructed test set, the datasets used in our simulations are all derived from the Essen folksong collection (Schaffrath and Huron, 1995). The Essen folksong collection is a corpus consisting of monophonic transcriptions of folksongs, originating from various geographical regions across the globe. The majority of the folksongs in this dataset originate from regions in Germany and China. We use a version of the Essen folksong collection encoded in humdrum format, which we obtained from http://kernscores.stanford.edu.

Folksongs without an annotated time signature, or with multiple time signatures are filtered out. The simulations described below use different subsets of this filtered version of the Essen folksong collection.

### 3.3. Classification performance and the influence of preceding context

The first expectation formulated in Section 2.5 concerns the model's ability to infer meters that agree with time signatures notated in scores. To evaluate this, classification performance is measured using ten-fold cross validation on a dataset of German folksongs. In a cross validation scheme, a model is trained and evaluated ten times on different partitions of the dataset into a training set and a test set. Reported classification scores are based on the average classification score over all ten partitions.

The second expectation we formulated is that models exploiting sequential probabilistic properties will perform better in this task than a similar model that does not exploit such properties. To evaluate this, we measure classification performance of five different models configured with order-bounds ranging from zero to four using cross validation. The order-bound parameter (see Section 2.2) allows us to vary the degree to which the model can learn sequential probabilistic properties of rhythms, interpolating between a model that can only learn distributions of onsets over metrical positions (order-bound zero) and a model that predicts the subsequent metrical position based on the metrical positions of the last four events (order-bound four).

The result of performing inference on the generative model—inferring meter from a rhythm—is not a single classification, but a posterior probability distribution over metrical interpretations. To determine in which meter the model interprets a rhythm, an additional inferential step is required. All classification scores reported in this paper are based on the interpretations with the highest posterior probability after observing the entire rhythm. An interpretation is considered correct if its phase and category agree with the annotated time signature.

**Figure 2:**
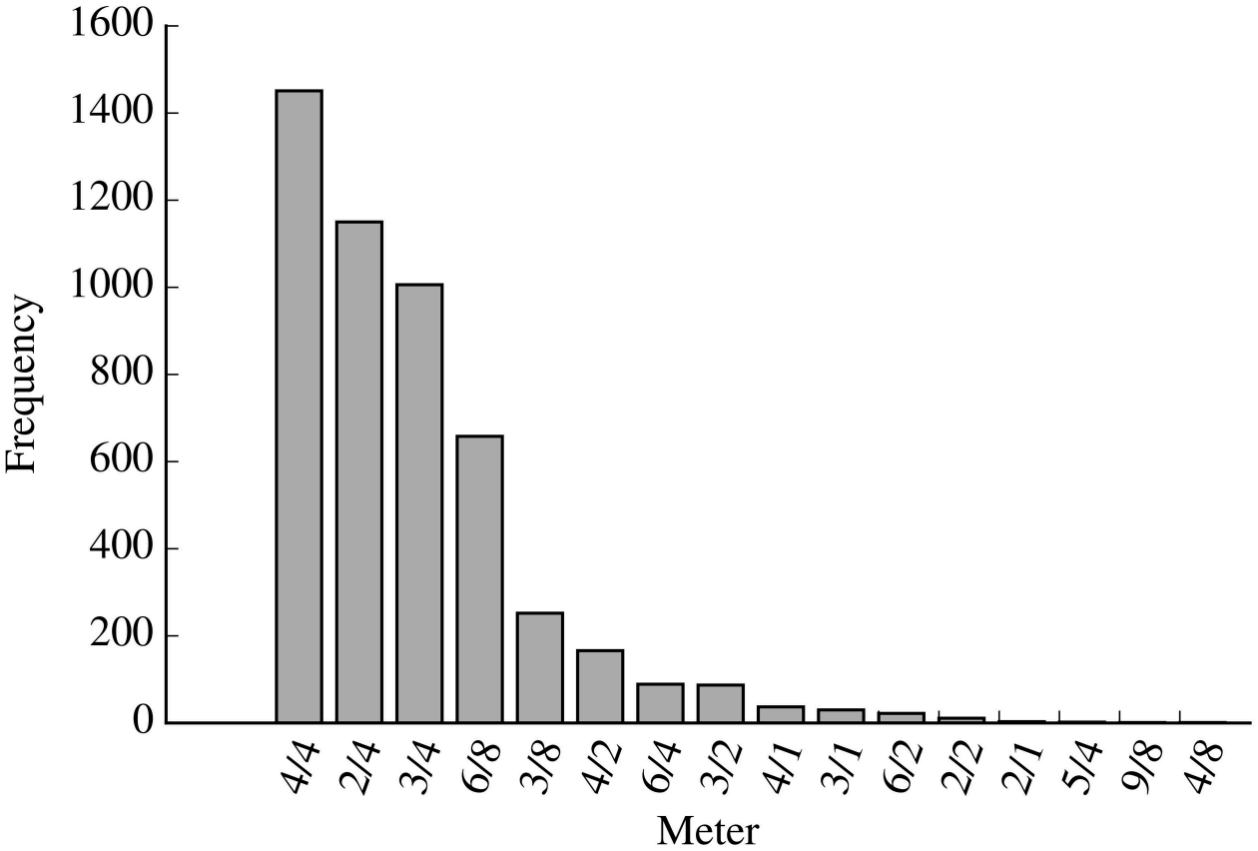
**Histogram showing the of the distribution of meters in the dataset of 4,966 German folksongs from the Essen folksong collection**.

For these simulations, we used rhythms extracted from 4,966 German folksongs in the Essen folksong collection. This set is constructed by selecting all melodies with an “ARE” record (area of origin; Huron, 1999) indicating a region of Germany from the Essen folksong collection, subject to the constraints described in Section 3.2. Figure 2 shows the distribution of meters in the resulting dataset. The most frequently appearing time signatures in this set are 4/4, 2/4, 3/4, and 6/8.

### 3.4. Does metrical inference reduce prediction error?

The third expectation we formulated is that a model using inferred meter to predict the onsets of musical events will outperform comparable models that do not use metrical inference. To assess whether metrical inference increases predictive performance we compare the model an IDyOM model that predicts event onset time without inferring meter. Prediction performance is measured by looking at average information content (see Section 2.4), which represents the discrepancy between predicted and observed events.

This IDyOM model is configured to use a single viewpoint, encoding inter-onset intervals between subsequent events, to predict onset time. Inter-onset interval is defined as the difference between the onset time of the final and penultimate event. Both models are trained and evaluated on the same dataset using cross-validation, and the input of both models consists only of onset times encoded in the event representation.

The results are reported, as before, for order-bounds varying from zero to four. The values represent average information content over cross validation folds.

### 3.5. Simulating enculturation

The fourth expectation concerning the model's behavior we formulated is that it should show sensitivity to the statistical properties of its training data. To investigate this, two types of statistical aspects of training data that affect the model's behavior in different ways are distinguished. The first aspect is the distribution of metrical categories in the training rhythms. This distribution is directly reflected in the prior distribution, encoding a priori likelihood of different metrical categories. The effect of the prior distribution on the model's behavior can be seen as *inferential biases*. The second aspect concerns the sequential structure of the training rhythms themselves. This aspect includes the distribution of onsets over different metrical positions, but also the typical unfolding of rhythms interpreted in a specific meter and the presence of stereotypical rhythmic patterns.

These two aspects of training data may influence the encountered prediction error on novel rhythms as well as the metrical category in which rhythms are interpreted. To investigate the effect of inferential biases, we focus on consequences of inferential biases for metrical interpretation. In the investigation of the statistical properties of rhythms themselves we focus on the effects of training data on prediction error.

The simulations described below are all conducted using an order-bound of four, since the cross validation results indicate that, out of the considered order-bounds, four works best (see Section 4).

#### 3.5.1. Inferential biases

A high prevalence of certain metrical categories in the music to which a listener has been exposed to previously may lead to inferential biases: a tendency to interpret rhythms in the pervasive category. In probabilistic terms, this is a sensible behavior: in the presence of uncertainty, it is optimal to tend toward categories with a high *a priori* likelihood of occurring. Such likelihoods are represented in the prior distribution over metrical categories. Inferential biases are top-down in the sense that they are independent of the particular rhythm encountered by the model. Once the model begins to process a rhythm, the prior distribution is updated by bottom-up evidence from the rhythm. Inferential biases can alternatively be understood as changing the initial state of meter induction. Meters favored by the prior distribution require less evidence from rhythmic events to gain a high posterior likelihood. In cases where a rhythm is ambiguous (i.e., provides evidence for two or more metrical categories), inferential biases toward either category can be decisive in the model's interpretation.

To investigate the effect of inferential biases, we train two models on a subset of the German folksongs described in Section 3.3 containing 658 2/4 (a simple duple meter), 658 3/4 (a simple triple meter) and 658 6/8 (a compound duple meter) training examples. We bias the prior distribution of one model to favor 3/4 interpretations while the other model is biased to favor 6/8 interpretations.

In this simulation the prior distribution is not estimated empirically using the relative frequency of metrical categories in the training data. Instead, the parameters of the prior distribution are manually set to the values shown in Table 1. The rationale behind this choice is that if we would manipulate the prior distribution by altering the number of training rhythms in a metrical category, the number of training examples from which the model predictive model of that category is learned would be affected, which introduces performance differences that cannot be attributed solely to the prior distribution.

**Table 1:** **Prior probabilities of metrical categories used for simulating inferential biases**.

**Category**	**Prior probability**	
	**3/4 biased**	**6/8 biased**
2/4	4/9	4/9
3/4	4/9	1/9
6/8	1/9	4/9

The consequences of the biased prior distribution are investigated using an artificially constructed test set. To construct this set, first, a set of rhythmic patterns is constructed by generating all possible patterns within the following constraints: the total duration of a pattern is exactly twelve sixteenth notes, none of the patterns begin with a rest and the minimum inter-onset interval is a sixteenth note. The resulting set consists of 2^11^ rhythmic patterns: each pattern begins with an onset and each sixteenth-note time point between the second and twelfth sixteenth-note can contain an event onset. Because twelve sixteenth notes is exactly the duration of one 3/4 or 6/8 bar, this set contains all rhythms with a minimum interval of a sixteenth note that fit in one bar of a 3/4 or 6/8 meter. To construct the final test set, each of these patterns is repeated four times. The repetition allows the model more time to converge on a single interpretation.

Both models are used to infer meter for each rhythm in the test set. Note that while three different categories, 2/4, 3/4, and 6/8, are considered, the quadruple repetition of patterns with a duration of twelve sixteenth notes may favor 3/4 and 6/8 interpretations. Since this potential bias is a property of the test set on which both models are evaluated, it does not cause problems for the evaluation of the effect of inferential biases.

We expect that inferential biases will increase the number of rhythms interpreted in the category corresponding to the bias. Due to the juxtaposition of 3/4 and 6/8 inferential biases, and the bar-level period-correspondence between these two meters, we expect to find the greatest degree of disagreement in interpretation of rhythms in the test set between the 3/4 and 6/8 categories: the 3/4 biased model will likely interpret rhythms classified by the 6/8 biased model as 6/8 in 3/4 and vice versa.

It seems plausible that 3/4 and 6/8 inferential biases will lead to some disagreement about the 2/4 category. An inferential bias may lead a model to interpret rhythms classified by the other model as 2/4 in the category corresponding to its bias. At the tactus level, 2/4 and 3/4 exhibit structural similarities: by convention, 2/4 and 3/4 both imply simple meters, where beats are subdivided into two smaller units. The 6/8 time signature, on the other hand, implies a compound meter. These (music-theoretic) similarities between 2/4 and 3/4 may lead the 3/4 biased model to interpret more rhythms, interpreted in 2/4 by the 6/8 biased model, according to its bias than the 6/8 biased model will out of the rhythms interpreted in 2/4 by the 3/4 biased model. It is worth noting that 2/4 and 6/8 have a different structural similarity at the level above the tactus: they are both duple meters. However, the duration of beat in 2/4 and 6/8, in our quantized input representation, is different, preventing this similarity from playing a role in our model.

The set of rhythms interpreted differently by both models likely consists of rhythms that do not strongly imply one specific interpretation. We expect such rhythms to be either ambiguous, or metrically over- or under-determined (London, 2012, pp. 75–76). Because we define a classification as the interpretation with the maximum posterior probability, the model always produces an interpretation of a rhythm, even if evidence from the rhythm is weak or conflicting. Therefore, some of the rhythms about which the models disagree may be metrically vague, i.e., not strongly suggesting any interpretation.

#### 3.5.2. Cultural distance between Chinese and German rhythms

In two simulations, we investigate how the model responds to being trained on folksongs originating from China or Germany. Music from these two areas might be different enough to lead to differences in rhythmic processing between enculturated individuals. By training the model on a dataset of Chinese and German folksongs, we can simulate how, according to the model, exposure to these stylistically different sets of rhythms affects perception.

To this end, we use two dataset sets, containing folksongs originating respectively from Germany and China. The German dataset is the same one that is used for the cross validation simulations described in Section 3.3. The dataset of Chinese folksongs is constructed in the same way as the German dataset, namely by selecting all folksongs from the Essen folksong collection whose “ARE” reference record (Huron, 1999) indicated a region in China and after first filtering out folksongs with zero or more than one annotated time signatures.

We run simulations in two separate conditions. In both conditions, two models are trained: one on a Chinese training set, and one on a German training set. Both of these models are subsequently evaluated on a separate Chinese and German test set consisting of rhythms that do not occur in the training data. In contrast to the simulation described above, we estimate the prior distribution in its normal way (see Equations 3 and 4).

The number of rhythms of each metrical category used in the test and train sets in the first and second condition are shown in Table 2.

**Table 2:** **Number of rhythms in different metrical categories in the training and test sets used in the simulation of enculturation**.

**Distribution of meters**		**Identical**				**Empirical**			
**Country of origin**		**Germany**		**China**		**Germany**		**China**	
**Dataset**		**Training**	**Test**	**Training**	**Test**	**Training**	**Test**	**Training**	**Test**
Meter	2/4	950	200	950	200	339	60	1,009	178
	4/4	132	0	132	0	427	75	90	16
	3/4	35	0	35	0	296	52	24	4
	3/8	19	0	19	0	74	13	13	2
	All	1,136	200	1,136	200	1,136	200	1,136	200

In the first condition (see the columns under “identical” in Table 2), we control for the effect of the prior distribution and use identical distributions of metrical categories in the training data of both models. This allows us to attribute observed effects to differences in the statistical properties of rhythms, ruling out effects of differences in the number of training examples or the differences in prior distributions. Meters considered in the simulation need to be well represented in both datasets. In the German and Chinese dataset that we have available, this constraint leaves 2/4, 3/4, and 3/8 as suitable categories. Despite this reduction, the number of rhythms in meters other than 2/4 in the Chinese dataset remains rather small.

Due to the small number of rhythms in meters other than 2/4 in the Chinese dataset, it is not possible to use a uniform distribution of meters in the test sets for this condition. Instead, we only include rhythms in 2/4 in the German and Chinese test set.

In the second condition (see the columns under “empirical” in Table 2), we allow the prior distribution to influence results and use empirical distributions of metrical categories in the training data of both models. By empirical, we mean that the relative frequencies of meters in the test and training sets that we used are equal to those observed in the Essen folksong collection. Both training sets contained in total an equal number of training examples.

Rhythms in the test sets for this condition are distributed to the same proportions as in the corresponding training sets. The Chinese test set predominantly contains rhythms annotated in 2/4 while the German test set also contains substantial numbers of rhythms in 3/4 and 4/4.

We expect that, on the Chinese and the German test sets, the model trained and tested on culturally similar music will exhibit lower average information content and higher classification performance than the model trained on culturally different music. We expect to see this pattern of results both for the identical, as well as for the empirical distribution of meters in the training data.

## 4. Results

### 4.1. Classification performance and preceding context

**Figure 3:**
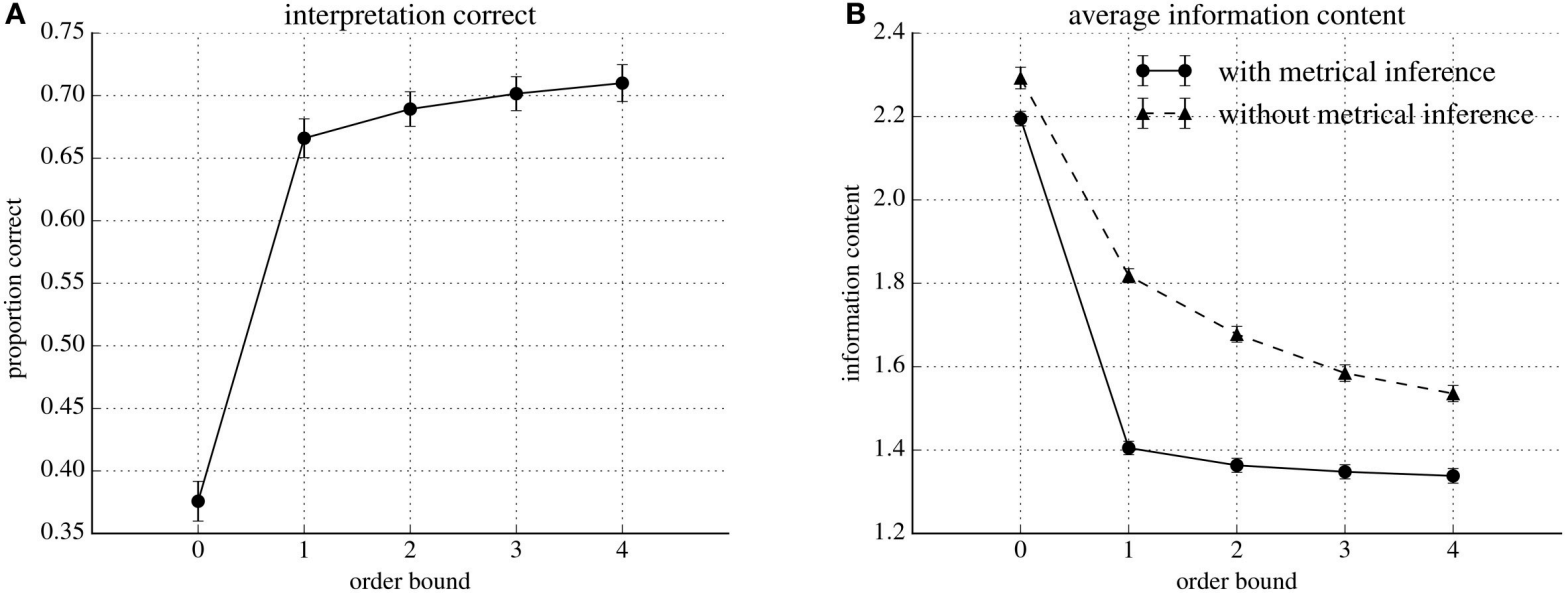
**Classification performance and average information content for five different models varying in order-bound, evaluated using ten-fold cross-validation**. Markers represent values obtained by averaging over the ten folds. Error bars extend one standard deviation above and below the average values. **(A)** Proportions of correctly classified interpretations. **(B)** Average information contents for the model (with metrical inference) compared to IDyOM without metrical inference.

Figure 3A shows the average number of correct interpretations found by our model at order-bounds ranging from zero to four. The averages are obtained by first averaging all per-event information contents (see Section 2.4) in the test set of one cross validation fold, and subsequently over all cross-validation folds. The standard deviations are calculated over the averages per cross validation fold. At order-bound zero, the model interprets rhythms in agreement with annotated time signatures in, on average 38%, of the cases. At order-bound one, classification performance increases sharply to, on average, 67% of the rhythms in agreement with the annotated time signature. Increasing order-bound further yields modest improvements. At order-bound four, the highest we tested, on average, 71% the rhythms were interpreted in agreement with the annotated time signature.

Variability in performance between different partitions of the data in a training and test set is low, as the small error bars in Figure 3A show.

### 4.2. Metrical inference and prediction error

Figure 3B shows prediction performance in terms of average per-event information content of rhythms under IDyOM (without metrical inference) and our extended version of IDyOM (with metrical inference). Both models were tested at order-bounds ranging from zero to four.

The results shows that, in general, information content decreases as order-bound increases for both the IDyOM model (without metrical inference) and our model (with metrical inference). The results also show that for all tested order-bounds, the average information content is lower our model (with metrical inference): for example 2.19 compared to 2.29 for order-bound zero and 1.34 compared to 1.54 at order-bound four.

### 4.3. Simulating enculturation

#### 4.3.1. Inferential biases

The results obtained from contrasting two models with manually manipulated prior distributions on an artificially generated test set are summarized in Table 3.

**Table 3:** **A contingency table showing the number of time signature classifications by a 3/4 biased model and a 6/8 biased model**.

		**3/4 biased**			
		**6/8**	**3/4**	**2/4**	**All**
6/8 biased	6/8	471	83	40	594
	3/4	0	395	0	395
	2/4	0	54	1,005	1,059
	All	471	532	1,045	2,048

The results shows that both models interpret approximately half of all rhythms in 2/4. The rightmost column shows that the 6/8 biased model interprets more rhythms in 6/8 than in 3/4, while the bottom row shows that the 3/4 biased model interprets more rhythms in 3/4 than in 6/8.

The numbers on the diagonal show that both models agree on the vast majority of interpretations. Both models agree on the interpretation of rhythms that are classified as 3/4 or 6/8 *despite* inferential bias: None of the rhythms that the 3/4 biased model interprets as 6/8 are interpreted differently by the 6/8 biased model. Similarly, none of the rhythms that the 6/8 biased model interprets as 3/4 are classified differently by the 3/4 biased model.

The numbers off the diagonal show that the greatest degree of disagreement occurs between the 6/8 and 3/4 categories, but there is also substantial disagreement between 2/4 and 3/4 and 2/4 and 6/8.

There are two categories of rhythms sensitive to inferential biases: The first category consists of 83 rhythms that the 6/8 biased model interprets in 6/8 while the 3/4 biased model interprets them in 3/4. The second category consists of rhythms that one model interprets in 2/4 while the other model interprets them in the category its biased toward. The 6/8 biased model interprets 40 rhythms in 6/8 that the 3/4 biased model interprets in 2/4. Out of the rhythms classified by the 6/8 biased model as 2/4, the 3/4 biased model interprets slightly more rhythms in agreement with its bias (namely 54), than the 6/8 biased model does out of the rhythms classified by the 3/4 biased model as 2/4 (namely 40).

#### 4.3.2. Cultural distance between Chinese and German rhythms

Table 4 shows average information content and classification performance obtained in the simulations of enculturation with German or Chinese folksongs. Results from two conditions are reported: one in which the German and Chinese training sets have an identical distribution of metrical categories and one in which they have empirical distributions of metrical categories.

**Table 4:** **Average information content and classification performance of models trained and evaluated on test sets with rhythms from Germany and China**.

	**Training set**	**Test set**			
		**Identical priors**		**Empirical priors**	
		**German**	**Chinese**	**German**	**Chinese**
Information content	German	1.21	1.63	1.34	1.72
	Chinese	1.32	1.49	1.70	1.49
Classification	German	0.84	0.80	0.73	0.72
	Chinese	0.59	0.77	0.47	0.75

*Results are reported for two different conditions. One in which training sets contain identical distributions of metrical categories, and one in which training sets contain empirical distributions of metrical categories*.

In both conditions the results can be said to show effects of enculturation: The average information content for models evaluated on rhythms from the same country as the rhythms in their training data (culturally familiar) is lower than for models trained on rhythms from the other country (culturally unfamiliar). Classification performance shows a similar pattern: in most cases, classification performance is better for models evaluated on culturally familiar rhythms. However, in the identical prior condition, classification performance of the German model on the Chinese test set was slightly higher than of the Chinese model. Furthermore, in the identical prior condition, the average information content of the Chinese model is lower when evaluated on the German test set compared to the Chinese test set.

For both models and in both conditions, but most notably in the identical priors condition, information content of rhythms in the Chinese test set was slightly higher than that of rhythms in the German test set.

**Figure 4:**
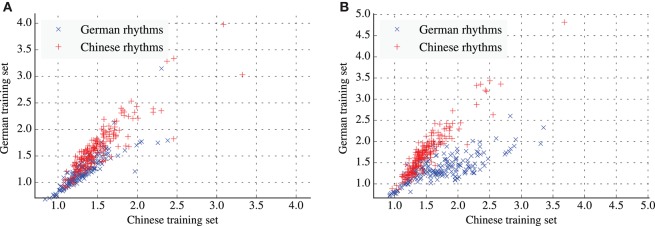
**Scatter plots of the average information content of rhythms for the Chinese and German models. (A)** Results for the training and test sets with fixed distributions of meters. **(B)** Results for the training and test sets with empirical distributions of meters.

Figures 4A,B project the rhythms from both test tests onto a two-dimensional plane. The coordinates of each rhythm are determined by the average information content of events in the rhythm under the Chinese model (x-axis) and German model (y-axis). Under this projection, rhythms from the two cultures form clusters that are to some degree spatially separated. The degree of separation is stronger in the empirical prior condition (Figure 4B). For both conditions, average information content of events in a single test set is highly correlated between both models (see Table 5).

**Table 5:** **Pearson product-moment correlation coefficients between average information content per rhythm under the German and the Chinese model, showing the degree to which information-content assigned to the same rhythms by both models is related**.

	**German test set**	**Chinese test set**
Fixed prior	0.74	0.94
Empirical prior	0.86	0.89

## 5. Discussion

A predictive coding view of perception entails that perception depends on generative models in the mind of the perceiver that are tuned by statistical properties of the environment, through evolutionary adaptation and sensory experience, to predict sensations. We hypothesized that effects of enculturation on the perception of meter can be understood in terms of predictive coding. To explore the consequences of this idea, we presented a probabilistic model of meter perception for which predictive coding served as the conceptual basis. The underlying hypothesis is that meter perception is the result of a strategy, based on statistical learning, probabilistic prediction and inference, for increasing predictive accuracy in processing of temporal events in music.

A set of expectations concerning the model's behavior was derived based on: the relevance of the model as a cognitive model of meter perception, theoretical proposals about the relation between rhythm and meter, the model's ability to reduce prediction error, and finally the model's potential to simulate enculturation. To investigate the degree to which the model meets these expectations, we ran a series of simulations. The results show that the model can infer metrical structure from rhythms, and that this ability improves when statistical properties of the succession of onsets in the metrical context are taken into account. A comparison with a similar model that does not use metrical inference demonstrates that metrical inference reduces prediction error in predicting the timing of musical events. Finally the results show hypothesized patterns of enculturation when models are trained on corpora varying, both naturally and artificially, in terms of distribution of meters and rhythmic properties.

The following sections discuss the simulation results in detail.

### 5.1. Meter classification and preceding context

A model of meter perception can reasonably be expected to interpret a simple rhythm in a meter that agrees with the time signature that an educated listener would use when transcribing that rhythm. The used rhythms were taken from folksongs in the Essen folksong collection (Schaffrath and Huron, 1995). Despite its possible relevance to determining the time signature, melodic information was disregarded. This limitation notwithstanding, cross-validation results indicate that the model generally infers interpretations that agree both in category and phase with annotated time signatures. The best performing model configuration interprets rhythms in a time signature and phase that agrees with annotations in the Essen folksong collection in 71% of the cases. These classifications were selected by the model out of a large pool of alternatives. Summing the number of possible phases per considered metrical category (see Section 2) yields 320 possible metrical interpretations. Many of these categories occur very infrequently in the training data, resulting in a low *a priori* likelihood for these categories. If we limit interpretations to the four most frequently occurring metrical categories—4/4, 2/4, 3/4, and 6/8—the number of interpretation options reduces to 48.

By varying the model's order-bound (the amount of preceding events that inform the prediction of the next event, see Section 2.2), we investigated to what degree learning statistical properties of the succession of metrical positions in rhythms improved the model's performance.

Increasing the order-bound from zero to one yields the most significant improvement in classification performance. This finding is consistent with results obtained by Temperley (2010) in a comparison of six onset-prediction models. Some of these models were metrical, which means they made use of provided (rather than probabilistically inferred) metrical information. Temperley (2010) found that out of the compared models, the two metrical and context-sensitive models, namely the first-order metrical duration model and hierarchical position model, yielded the lowest cross-entropy (information content) score.

**Figure 5:**
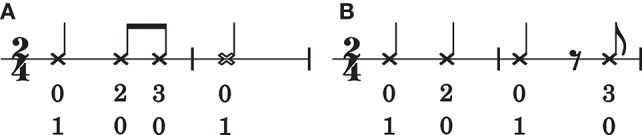
**Two rhythms that result in different orderings of the same set of **mp** ⊗ **bd** viewpoint elements**. The number-pairs below the notes are the values of mp ⊗ bd. The top number represents the value of the bd (bar-distance) viewpoint, the bottom number represents the value of the mp (metrical position; expressed in multiples of an eighth note duration) viewpoint. The rhythm in **(A)** is structurally different from the rhythm in **(B)**, but under a zeroth-order mp ⊗bd viewpoint, they are indistinguishable.

The performance increase between order bound zero and one is unsurprising. In a zeroth-order model, events in a rhythm are conditionally independent given a meter. If the meter is known, the probability of the next event only depends on its metrical status and is independent of preceding events^1^. In a zeroth-order model, a rhythm is a “bag of notes”: the order in which notes occur is irrelevant to the final outcome. However, note-order bears consequences for the metrical interpretation of a rhythm, as illustrated in Figure 5. The rhythm in Figure 5A is structurally different from the rhythm in Figure 5B, yet under a zeroth-order model using mp ⊗ bd metrical viewpoints (see Section 2.3) these rhythms are indistinguishable.

The results show that classification and prediction performance, increases further when order-bound is increased to four. Since this improvement is relatively modest, it remains to be seen to what extent probabilistic information about the succession of multiple events facilitates metrical inference. Perhaps the effect of order-bound would be more pronounced for music styles with more complex rhythms than the folksongs used here.

### 5.2. Metrical inference reduces prediction error

We proposed that meter perception may result from predictive coding: interpreting onsets in a rhythm as the result of a generative model with different periodic categories (meters), that are inferred from the pattern of onsets itself, may facilitate prediction of future onsets. Interpreting a rhythm in a metrical framework allows a listener to relate the observed events to patterns they observed previously. A computational probabilistic model that infers meter to predict the timing of events, such as the one presented here, should therefore encounter a lower prediction error in empirical rhythms compared to a similar model that does not infer meter.

To evaluate this, we compared prediction performance of the presented model to an IDyOM model that predicts the event onset times without using metrical inference. This comparison seems natural because the presented model implements metrical inference directly on top of IDyOM as explained in Section 2.

Simulations show that the meter inferring model reduces prediction error compared to IDyOM (without metrical inference) under all tested order-bounds. These results support the suggestion that inferring meter may improve temporal prediction of events in rhythms.

### 5.3. Simulating enculturation

The goals of the simulations concerning enculturation were to investigate how our model's behavior is shaped by the statistical properties of rhythms in its training data, and to investigate the extent to which these statistical properties can be exploited to improve the prediction and metrical interpretation of stylistically similar rhythms. We first explored the consequences of inferential biases on an artificially constructed set of potentially ambiguous rhythms. Then, we studied the effect of statistical properties of sets of rhythms on metrical inference. The results show that when tested on Chinese rhythms, models trained on rhythms of Chinese folksongs show better prediction performance than models trained on German folksongs. The converse was true when the models were tested on German folksongs.

This simulation of enculturation should be seen as a proof-of-concept: Patterns of quantized onset times annotated with meter are a limited representation of the rich variety of musical and non-musical experiences that may shape listeners' perception of meter. In the musical domain, timbre, polyphony, expressive timing and dynamics are some examples of aspects not considered by our approach that all could plausibly form part of the experiences that shape meter perception. Nevertheless, it is possible that monophonic corpora of rhythms from different cultures can predict some enculturation effects. The methodology presented here is an illustration of how such predictions could be made.

#### 5.3.1. Inferential biases

Inferential biases were introduced into the model by directly manipulating the prior distribution, while avoiding differences in the amount of training examples per metrical category, which would influence the results.

We contrasted two models: one with a 6/8 inferential bias, another with a 3/4 inferential bias. The models were evaluated on an artificially constructed test set of rhythms with the potential for ambiguity between 3/4 and 6/8. These test rhythms were not annotated, as we intended find the set of rhythms for which inferential biases could swing the model's interpretation.

The results show that inferential biases affected the distribution of interpretations over metrical categories in ways that we expected: Each model interpreted more rhythms in the category corresponding to its bias than the other model. Both models agreed on the interpretation of the majority rhythms. These rhythms contained enough evidence toward a particular interpretation to override the model's inferential bias. As we expected on music theoretic grounds, the 3/4 biased model swung the interpretation of slightly more rhythms, interpreted in 2/4 by the 6/8 biased model, to a 3/4 interpretation than the 6/8 model did out of the set of rhythms interpreted in 2/4 by the 3/4 biased model.

**Figure 6:**
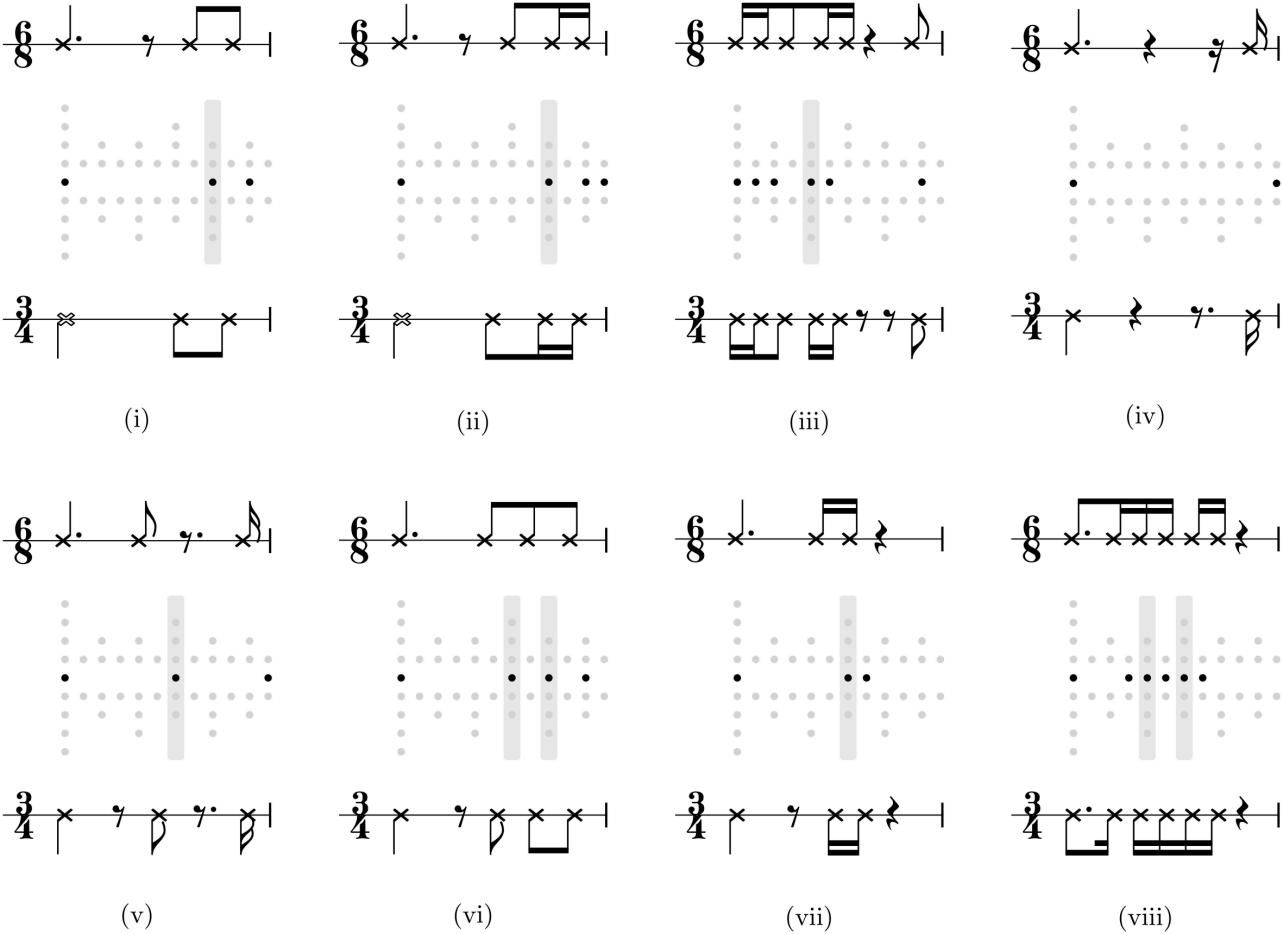
**The full set of rhythms interpreted as non-anacrustic 3/4 rhythm by a model with a 3/4 inferential bias and a non-anacrustic 6/8 rhythm by a model with a 6/8 inferential bias**. Each rhythm is shown in dot notation between a compound 6/8 metrical grid (above) and a 3/4 simple metrical grid (below). The gray bars highlight onsets that fall on beats with different theoretical saliencies in both interpretations. Score transcriptions of the rhythms in 6/8 and 3/4 are shown above and below the grids.

Eight rhythms interpreted were interpreted in 3/4 without pick up by the 3/4 biased model and in 6/8 without pick up by the 6/8 biased model. These rhythms are shown, by way of example, in Figure 6, along with metrical grids contrasting a simple 3/4 interpretation with a compound 6/8 interpretation. That the interpretation of these rhythms could be influenced depending on inferential bias of the model suggests that they are ambiguous (e.g., Figure 6vi), and/or metrically underdetermined (e.g., Figures 6i,iv), or metrically vague, i.e., not strongly suggesting any interpretation (e.g., Figure 6viii).

#### 5.3.2. Cultural distance between Chinese and German rhythms

In general agreement with the hypotheses presented in Section 2.5, the results in Table 4 show that models evaluated on a test set with rhythms from the same country as the rhythms they were trained on exhibit a lower average per-event information content. The classification scores for models trained on culturally familiar rhythms were also higher compared to models trained on culturally unfamiliar rhythms, except on the Chinese test set in the identical prior scenario. It could be that rhythms in the Chinese portion of the Essen folksong collection (Schaffrath and Huron, 1995) were less consistently annotated, but further investigation is necessary to determine whether this is the case. The pattern of results suggests that the statistical properties of Chinese and German rhythms are different, and that these differences can be exploited to optimize prediction and metrical inference on rhythms from one of the countries.

In a recent study comparing recognition memory in North American listeners on Turkish classical music and Western art music, Demorest et al. (2016) found that rhythmic properties of music did not contribute to an enculturation effect on memory performance. At a first sight, these results seem surprising in the light of earlier studies that did find effects of enculturation related to rhythmic organization of music (Hannon and Trehub, 2005a,b; Hannon et al., 2012). However, it is possible that while rhythms are capable of eliciting effects of enculturation, such rhythms did not occur in the stimuli used by Demorest et al. (2016). Demorest et al. (2016) used a small set of stimuli that were not specifically selected to contain rhythms likely to elicit an effect of enculturation. The methodology applied in this simulation of enculturation is an example of how probabilistic models of rhythm perception can be employed to predict which rhythms are likely to elicit an effect of enculturation.

We hypothesized that fine-tuning of perception to the statistical properties of musical rhythms in one's environment in a way that leads to a reduction of prediction-error in rhythms typical of one's environment leads to differences in the processing of meter. This idea is closely related to the notion of *cultural distance*—the degree to which pitch relations in a musical excerpt resemble the pitch relations typical to music from one's own culture—introduced recently by Demorest and Morrison (2016). The *cultural distance hypothesis* (Demorest and Morrison, 2016) states that cultural distance is predictive of various culturally dependent responses such as preference, tension, expectation, and memory. This hypothesis is supported by a series of studies where cultural distance of stimulus material was found to affect memory performance (for an extensive overview, see Morrison and Demorest, 2009). Demorest and Morrison (2016) propose that cultural distance could be measured using probabilistic models of melodic expectancy, such as IDyOM, that learn the statistical properties of music from a particular culture. Music that is culturally distant from the music such a model is trained on should be predicted less effectively than culturally familiar music. As such, in the context of a cross-cultural study, average information content—the degree to which observed events deviate from one's expectations—can be seen as an operational definition of cultural distance.

The model presented here can supplement predictions about *melodic* cultural distance as provided by existing probabilistic models, with predictions about *rhythmic* cultural distance. Cultural distance, as predicted by our probabilistic model, can then be read directly from Figures 4A,B. If the probabilistic aspects of rhythm learned by the presented model correspond to those implicitly learned by human listeners, then, according to the cultural distance hypothesis, rhythms in the top-right part of Figures 4A,B should be more difficult to remember for German listeners while rhythms in the bottom-right part of Figures 4A,B should be more difficult to remember for Chinese listeners.

Other culturally dependent responses mentioned by Demorest and Morrison (2016) such as, expectation, preference, and tension can be potentially linked to information content as well. Regarding expectation, information content is a direct consequence of predictive failure and has been shown to account well for human pitch expectations (Pearce, 2005; Hansen and Pearce, 2014). Regarding preference, perceived groove and experienced pleasure have been hypothesized to depend on the right balance between predictability and unpredictability (Witek et al., 2014). Furthermore, influential proposals have postulated close ties between expectation and both emotional responses to music (Huron, 2006) and musical meaning (Meyer, 1957). Regarding tension, melodic expectation has recently been linked to expressive performance, which in turn was linked to perceived tension (Gingras et al., 2015).

### 5.4. General discussion

While it is commonly assumed that the metrical accent of a beat, as derived from formal hierarchical descriptions of meter (Lerdahl and Jackendoff, 1983), is proportional to the probability of onsets at those beats, recent findings by Holzapfel (2015) and London et al. (2016) challenge this view. London et al. (2016) suggested that onset frequency need not be correlated with metrical accent for effective communication of meter. Instead, they argue, it is the recurrence and stability of rhythmic figures in the context of specific meters that may play a key role in the relation between rhythm and meter.

The results we presented show that models which take into account the preceding context of musical events, thus possessing the potential to learn the typical unfolding of multiple characteristic rhythmic patterns under different meters, are generally better at predicting rhythms and reconstructing annotated meters from note onsets alone. These findings, we would argue, provide further support for the idea the relationship between rhythm and meter is not only characterized by the distribution of note onsets, but also by characteristic rhythms and statistical properties of succession of interval between events.

The model we presented learns a generative model of rhythms from an annotated corpus. The supervised aspect of this approach challenges the cognitive plausibility of our model. Humans develop a feel for meter in their own culture without someone explicitly informing them about the “right” metrical interpretation. Nevertheless, situated exposure to rhythm almost always happens within a context containing an abundance of multi-sensory information related to the rhythmic practice. Within the music itself, other instruments, expressive timing and dynamics may provide strong metrical cues. In the environment, being rocked to music as an infant, participating in dancing or observing other people dance all contribute to the multi-sensory context by which rhythm perception is shaped. While not entirely putting concerns related to the supervised aspect of our approach to rest, metrical annotations in our training data can potentially be seen as capturing some of the information communicated in situated exposure to rhythms.

We have only considered event onset times in the present study, but other musical aspects such as melodic repetition are known influence the perception of meter as well (Hannon et al., 2004). A full account of meter perception should take these aspects into account. Our model could be a good starting point for such an account: due to the implementation of the model in IDyOM, it is possible to link metrical viewpoints with melodic viewpoints and incorporate melodic aspects into the generative model.

Another limitation of the current model is its relatively simple representation of metrical structure. Time signatures fall short in capturing the structural complexity of perceived meter. The model treats metrical categories as independent generative models and structural similarities between meters remain unexploited. The model is limited in its interpretation of rhythms into metrical categories by the categories observed in training data. In future work, we will seek to address these limitations by extending the model's representation of metrical structure.

The model introduced here represents an extension of previous work in probabilistic modeling of music (Conklin and Witten, 1995; Pearce, 2005). It is worth pointing out that the predictive mechanisms on which the model presented here is based, are domain independent (Pearce and Wiggins, 2004). The PPM^*^ sequence prediction methods we employ can be applied to any domain that can be represented as structured sequences of symbols. Indeed, they were originally proposed in the field of text compression, but have proven to be useful in cognitive models of melodic expectation as well (Pearce, 2005; Pearce and Wiggins, 2012).

In summary, we have presented a computational probabilistic model meter perception, grounded in a predictive coding perspective of perception. The model has the potential to simulate musical expectations resulting from the perception of meter, shaped by previous exposure. The results show that the model can interpret simple rhythms in meters that agree with annotated time signatures and that it generates the hypothesized effects of enculturation. Simulations such as the ones presented here, can be used to generate theoretical predictions for cross-cultural studies of rhythm perception. Future research will determine the extent to which the learning processes implemented by our model capture aspects of those at work in human listeners.

## Author contributions

BW designed and implemented the model. The model builds on IDyOM, which was designed and implemented by MP; MP and HH improved the model; HH, MP, and BW designed the simulations; BW performed the research and analyzed the data; BW wrote the paper; HH and MP improved the paper.

## Funding

HH is supported by a Distinguished Lorentz fellowship granted by the Lorentz Center for the Sciences and the Netherlands Institute for Advanced Study in the Humanities and Social Sciences (NIAS) and a Horizon grant of the Netherlands Organization for Scientific Research (NWO). BW and MP also received support from the EPSRC Digital Music Platform Grant held at Queen Mary (EP/K009559/1). MP is supported by a grant from the UK Engineering and Physical Science Research Council (EPSRC, EP/M000702/1).

### Conflict of interest statement

The authors declare that the research was conducted in the absence of any commercial or financial relationships that could be construed as a potential conflict of interest.

## Footnotes

^1^The bd viewpoint used in our simulations indirectly introduces minor context dependency: if its value zero it means that the current note is the first note in the bar.
